# Genome-wide identification, phylogeny and expression analysis of the *SPL* gene family in wheat

**DOI:** 10.1186/s12870-020-02576-0

**Published:** 2020-09-11

**Authors:** Ting Zhu, Yue Liu, Liting Ma, Xiaoying Wang, Dazhong Zhang, Yucui Han, Qin Ding, Lingjian Ma

**Affiliations:** 1 College of Agronomy, Northwest A&F University, Yangling, 712100 China; 2 College of Life Science, Wuhan University, Wuhan, 430072 China; 3 College of Horticulture, Northwest A&F University, Yangling, 712100 China

**Keywords:** *SPL* gene family, Phylogenetic analysis, Expression patterns analysis, Wheat

## Abstract

**Background:**

Members of the plant-specific *SPL* gene family (squamosa promoter-binding protein -like) contain the SBP conserved domain and are involved in the regulation of plant growth and development, including the development of plant flowers and plant epidermal hair, the plant stress response, and the synthesis of secondary metabolites. This family has been identified in various plants. However, there is no systematic analysis of the *SPL* gene family at the genome-wide level of wheat.

**Results:**

In this study, 56 putative *TaSPL* genes were identified using the comparative genomics method; we renamed them *TaSPL001* - *TaSPL056* on their chromosomal distribution. According to the un-rooted neighbor joining phylogenetic tree, gene structure and motif analyses, the 56 *TaSPL* genes were divided into 8 subgroups. A total of 81 *TaSPL* gene pairs were designated as arising from duplication events and 64 interacting protein branches were identified as involve in the protein interaction network. The expression patterns of 21 randomly selected *TaSPL* genes in different tissues (roots, stems, leaves and inflorescence) and under 4 treatments (abscisic acid, gibberellin, drought and salt) were detected by quantitative real-time polymerase chain reaction (qRT-PCR).

**Conclusions:**

The wheat genome contains 56 *TaSPL* genes and those in same subfamily share similar gene structure and motifs. *TaSPL* gene expansion occurred through segmental duplication events. Combining the results of transcriptional and qRT-PCR analyses, most of these *TaSPL* genes were found to regulate inflorescence and spike development. Additionally, we found that 13 *TaSPLs* were upregulated by abscisic acid, indicating that *TaSPL* genes play a positive role in the abscisic acid-mediated pathway of the seedling stage. This study provides comprehensive information on the *SPL* gene family of wheat and lays a solid foundation for elucidating the biological functions of *TaSPLs* and improvement of wheat yield.

## Background

Gene families, consisting of multiple genes that share similar structures and functions, act in important roles for a given organism. Now, all kinds of gene families have been found in eukaryotes, such as *bHLH* [1], *TCP* [2], and *Prx* [3]. Among all gene families, SPL (squamosa promoter binding protein, SBP) is a plant-specific family that is widely distributed in green plants. The plant SPL proteins, bind to the SQUAMOSA promoter of MADS-Box genes, were first identified in a cDNA library of the *Antirrhinum majus* inflorescence [4]. These proteins contain a specific SBP domain that comprises approximately 70 amino acids and possesses two zinc finger sites (Cys-Cys-His-Cys and Cys-Cys-Cys-His) [5, 6]. In this domain, the 4 amino acid residues coordinate one zinc ion, playing an important role in maintaining the stability of the protein’s configuration. Besides, a conserved nuclear localization signal located at the C-terminus of the SBP domain overlaps with the second zinc finger structure [7] and directs the protein to the nucleus for regulating the transcription of downstream genes.

In recent years, a large number of *SPL* genes have been successfully cloned in various plants, and these *SPL* genes distribute from *C. reinhardtii* to *P. patens* and higher plants [8]. At present, the identification and evolution of the *SPL* gene family have been studied in *A. thaliana* [9, 10], rice [5], soybean [11], *Populus* [12], *Petunia* [13], *Gossypium* [14], and tobacco [15]. Based on the analysis of sequence homology and phylogeny, this family is usually classed into 6–9 subgroups. Guo [8] reported that 120 *SPL* genes from nine species divided into 3 groups, all *SPL* genes from land plants have been classed into two distinct groups and 7 subgroups. Yang [5] divided 35 *SPLs* from rice and *A. thaliana* into three categories: A, B, and C, with all *SPL* genes classified into 9 subfamilies. In *Populus* [12], the 28 *PtSPLs* were separated into 8 subgroups. In *Salvia miltiorrhiza,* 15 *SPL* genes were divided into 6 subgroups [16]. Unfortunately, little research has been conducted to show the classification of the *SPL* gene family in wheat.

The expression levels of *SPL* genes can be controlled by miR156/157. Rhoades [17] and Schwab [18] found that 11 of 17 *SPLs* in *A. thaliana* contain miR156/157 recognition sites. Schwab [18] reported that overexpressing miR156 in *A. thaliana* downregulated the expression of multiple *SPLs*, however, *SPLs* without this recognition site were unaffected. After mutating the miR156/157 recognition site of *AtSPL3*, the transcription level of *AtSPL3* was significantly increased [19]. Salinas observed the complementary expression fashion in apices and stamen of *SlySPL* genes with miR156/157, revealing that *SlySPL* genes are regulated by miR156/157 [20].

The *SPL* genes, whose expression levels are governed by miR156/157, play critical roles in various aspects of plant growth and physiology, including plant embryos, tissues, and vegetative-phase changes, transduction of gibberellin and light signal, and drought stress and salt stress responses. For instance, Inhibiting post-translational modification of the AtSPL3 protein caused early flowering in *Arabidopsis* [19]. *AtSPL3*, *− 4*, and *− 5* regulated the expression of the key downstream genes *AtAP1*, *AtFUL*, and *AtLFY* to be involved in floral meristem development [10]. Zhang has shown that *SPL8* gene positively regulated GA signaling in the flower, however, negative roles were found in the seedling, implying that *AtSPL8* modulates GA signaling in plant development [21]. Wang purported that *OsSPL16* can be effectively improved the grain quality and yield, by increasing cell division and grain filling, implying that this gene has positive consequences for rice grain development [22]. *OsSPL14* inhibited the number of tillers in rice, but promote panicle branching to increase grain weight together with stronger stems [23]. At low temperature (5 °C), both *VvSBP3* and *VvSBP5* were upregulated but *VvSBP4* and *VvSBP7* were downregulated, indicating that *VvSBP3* and *VvSBP5* are associated with low-temperature responses in *Vitis vinifera L.* [24]. The *BpSPL9* respond to the salt and drought stress, Overexpressed the *BdSPL9* in birch leaves can scavenge of ROS to improve the tolerance of salt and drought stress [25]. Much is know about the functions of *SPL* genes in *A. thaliana* and rice, however, such information for wheat is limited.

The plant-specific *SPL* family plays an important role in growth and development. With the progress of genome-sequencing techniques, various transcription factors or gene families in plants have been identified. Although many plant *SPL* gene family members have been identified, this is not the case for wheat, and knowledge of *SPL* gene functions in this species is limited. In this study, we used bioinformatics methods to identify *SPL* genes in wheat, and we analyzed *TaSPL* characteristics based on the results of gene structures, motifs, cis-elements, phylogenetic relationships, gene duplications, GO annotation, protein-protein interactions, and gene expression patterns, and predicted their functions. Our results provided information on the functional elucidation and evolution of *SPL* genes in wheat.

## Results

### Genome-wide identification and chromosomal location of *TaSPL* genes

To obtain *TaSPL* genes from the whole genome of wheat, two methods, HMM and BLASTP, were used for identification, and two online websites, Pfam and CDD, were used for confirmation. Ultimately, 56 genes were designated *TaSPL* genes; all are unevenly distributed on the wheat chromosomes. The 56 putative *TaSPL* genes were renamed *TaSPL001* to *TaSPL056* based on chromosomal locations (Additional file 1: Table S1). Among them, 34% (19) mapped to the A and D genomes, respectively; 32% (18) located on the B genome. TaChr7A and TaChr7D contain a large number of *SPL* genes, but no *TaSPL* genes are located on chromosomes 4B and 4D.

The 56 SPL proteins were submitted to the cello website to predict their subcellular localizations; as shown in Table S1, all of the SPL proteins were localized to the nucleus, and 3 SPL (TaSPL034, TaSPL049, TaSPL052) also distribute to the plasma membrane, suggesting that the 56 *TaSPL* proteins perform their functions in the nucleus. The 56 predicted TaSPL proteins exhibit diversity in amino acid length and protein molecular weights (MWs). The MWs of the TaSPL proteins range from 20.11 to 123.69 kDa. The lengths of the TaSPL proteins vary from 192 to 1129 aa, with the longest protein being TaSPL043 and the shortest being TaSPL004, TaSPL007, and TaSPL010.

### Gene structure and motif compositions of the *SPL* gene family in wheat

**Fig. 1:**
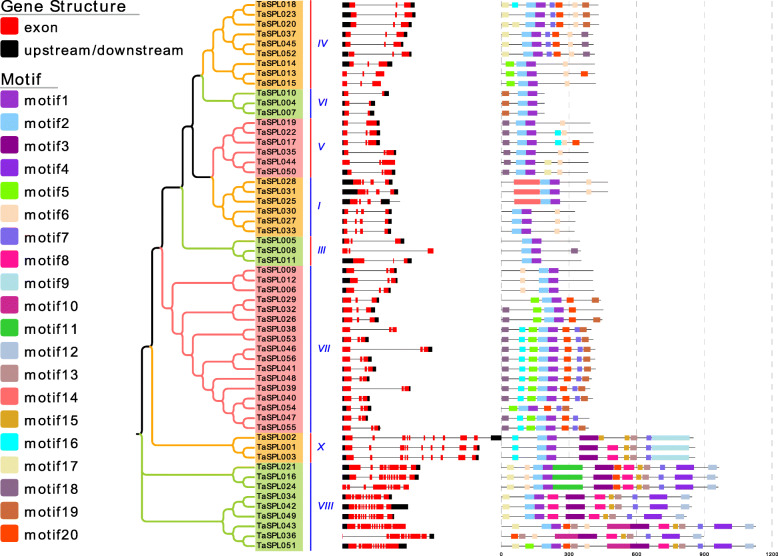
Gene structure and conserved motifs analysis of *TaSPL* genes based on phylogenetic relationship. Red boxes represent exon, and the black boxes represent upstream/downstream sequence. Each motif is represented by a colored box. The length of box corresponds to the motif length. The gene and protein length are indicated by the scale at bottom

Gene structure and motif diversity is a mechanism that promotes the evolution of gene families. We constructed a phylogenetic tree of *TaSPL* genes and analyzed the exons, introns, and conserved motifs (Fig. 1). The results showed that despite being different with regard to exon position, *TaSPL* genes from the same subfamily share a similar genetic structure, and the number of exons in most *TaSPL* genes is conserved. Approximately 59% (33) of the genes contain 3 exons, but 12 genes in subgroup VIII and subgroup X possess the largest number of exons, at 8–11. For example, *TaSPL001* in subfamily X contains 10 exons, and *TaSPL042* in subfamily VIII contains 11 exons.

We submitted the entire protein sequences of TaSPL to MEME software to detect the motif compositions and explore the motif diversity. Twenty specific motifs were defined and named motif 1 to 20. The TaSPL proteins exhibit similar conserved motif compositions, in particular, all TaSPL proteins except TaSPL036 contain motif 1 and motif 2, suggesting that these two motifs are important components for TaSPL protein sequences. Not only the common motifs exist in all SPL proteins, but also SPL members in separate subgroups also contain their specific motifs. For example, motif 18 is present in all SPL members of subfamily VII and subfamily IV; motif 20 is found in subfamily VII. In short, members in the same subfamily share similar gene structure and motif compositions, while different subgroups contain the specific structure, implying that the *TaSPL* gene family presents the functional conservation and diversity during evolution.

### Multiple sequence alignment, phylogenetic analysis of *TaSPLs*

**Fig. 2:**
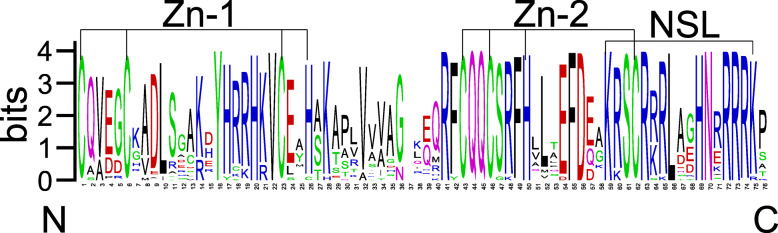
The SBP domain contained in TaSPL proteins. There are three conserved structures contained in the SBP domain: Cys-Cys-His-Cys, which represents the first zinc finger motif, and Cys-Cys-Cys-His, which represents the second zinc finger motif. One nuclear localization signal (NSL) is located at the C-terminus

Multiple sequence alignment of the full-length TaSPL proteins was carried out. The results showed that the specific SBP domain is conserved among the 56 TaSPLs (Fig. 2). Approximately 76 aa comprise an SBP domain that contains two zinc finger motifs with the structure Cys-Cys-Cys-His and Cys-Cys-His-Cys, and one nuclear localization signal (NSL) at the C-terminus. Three conserved sequences, CQQC, SCR, and RRR, appear in the TaSBP domain, except for TaSPL036, which does not possess the CQQC sequence.

**Fig. 3:**
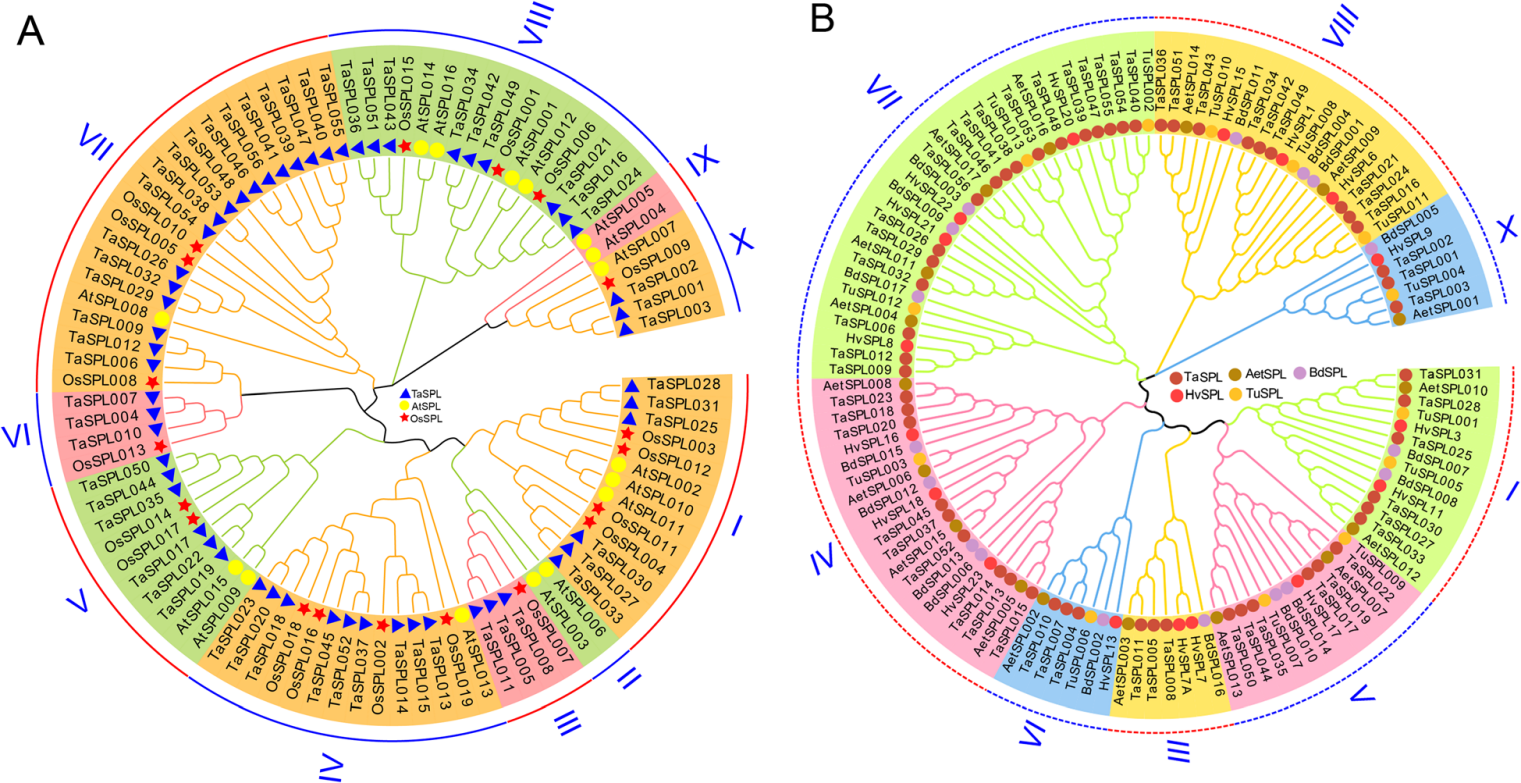
The phylogenetic tree of *SPL* genes in wheat. Phylogenetic analysis of *SPL* genes in wheat, *A. thaliana* and rice (**a**). Blue triangles represent *TaSPLs*, yellow circles *AtSPLs*, and red stars *OsSPLs*. Phylogenetic analysis of *SPL* genes in wheat, *B. distachyon*, *T.urartu*, *A. tauschii* and barley (**b**). Dark red circles represent *TaSPLs*, brown circles *AetSPLs*, red circles *HvSPLs*, yellow circles *TuSPLs*, and purple circles are *BdSPLs*

To evaluate the evolutionary relationships of *SPL* genes in wheat, rice, and *A. thaliana*, a neighbor-joining phylogenetic tree was constructed using the full-length SPL proteins (Fig. 3A). Ninety-one *SPL* genes from the above three species were divided into 10 subfamilies (I to X). According to this analysis, the *SPL* family of wheat is closer to *SPL* genes in rice than *A. thaliana*. Except for subfamilies II, III, VI, and IX, the remaining subfamilies contain 3 species *SPL* genes. For example, subfamily I contains 3 *AtSPLs*, 4 *OsSPLs*, and 6 *TaSPLs*. 2 *AtSPLs*, 2 *OsSPLs*, and 6 *TaSPLs* comprise subfamily V. This indicates that *TaSPL* genes did not evolve with the characteristics of monocotyledons and dicotyledons, and that the *SPL* gene family was formed before the differentiation of these two classes of the plant. We also found 22 paralogous gene pairs in the NJ-tree, among them, 4 gene pairs are from *A. thaliana* and 18 genes from wheat, indicating that the *TaSPL* genes expanded in a species-specific manner.

The same methods were used to identify 17, 13, and 17 *SPL* genes from *B. distachyon*, *T.urartu*, and *A. tauschii* genomes and downloaded the 17 *SPL* gene sequences of barley [26]. Then we constructed a phylogenetic tree (Fig. 3B). The 120 *SPL* genes from 5 species were still divided into 8 subgroups. We detected 13 orthologous *SPL* gene pairs from wheat and *A. tauschii*, 3 from wheat and *T.urartu*, and 1 from wheat and barley. Conversely, no orthologous gene pairs were found in wheat and *B. distachyon*. It is implied that the *TaSPL* genes share a strong evolutionary relationship with *AetSPLs* and *TuSPLs*.

### Gene duplication and synteny analysis of *TaSPL* genes

**Fig. 4:**
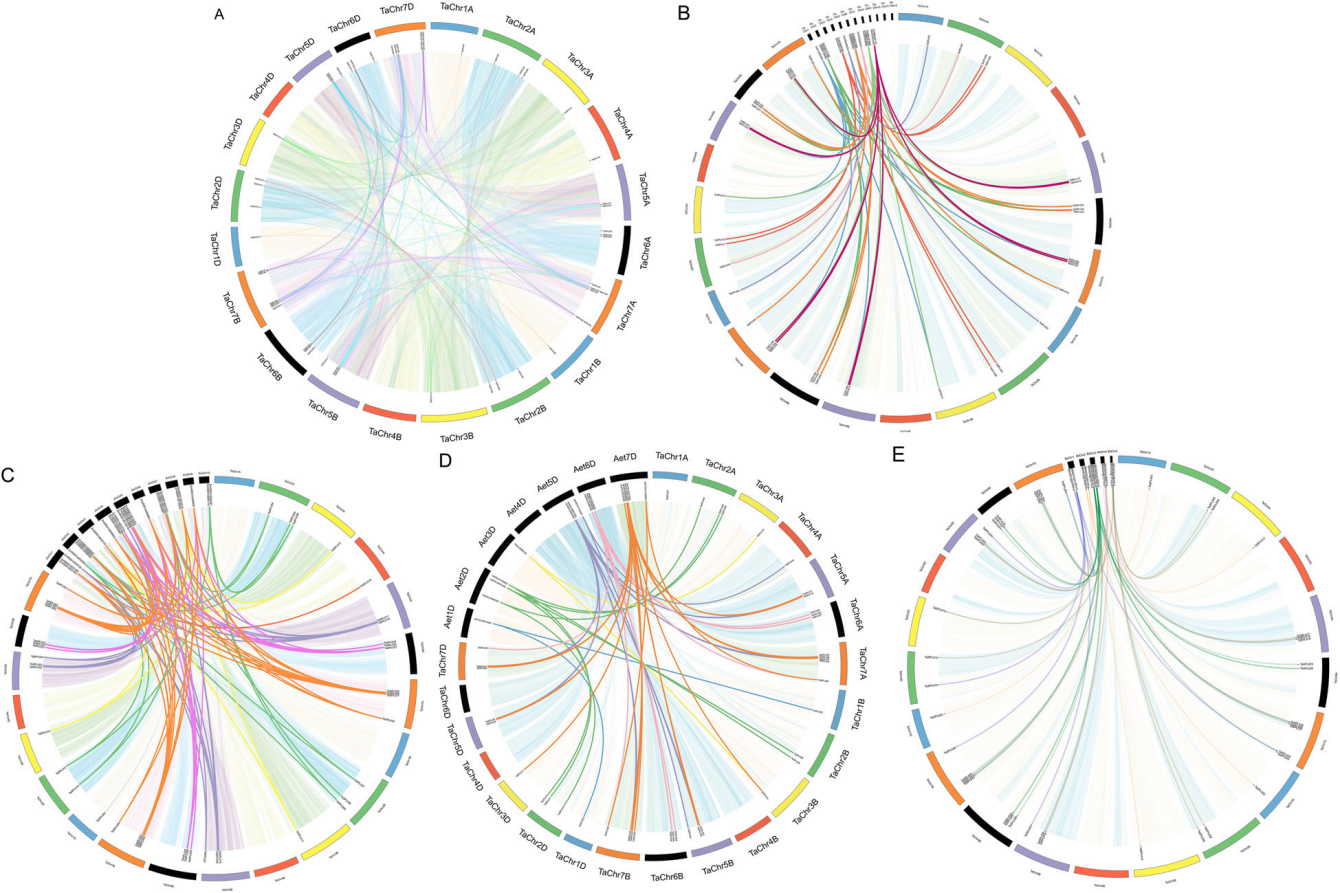
Colinearity analysis of *TaSPL* genes. Duplicated gene pairs in the wheat genome (**a**); colinearity analysis of *TaSPL* genes with rice (**b**), maize (**c**), *A. tauschi* (**d**), and *B. distachyon* (**e**). The light background represents synteny blocks in the whole genome of the five species, and dark lines represent collinear gene pairs of *TaSPLs*

Gene duplication, an indispensable mechanism, can expand new genes that share similar or different functions; therefore, we analyzed the duplication events that occurred in the *TaSPL* gene family. Eighty-one *SPL* gene pairs from wheat were detected as duplicated gene pairs (Fig. 4A, Additional file 1: Table S3), with 8 being tandem duplications, and 73 were associated with a segmental duplication event. The number of segmental duplication events in the *TaSPL* gene family was found to be higher than that of tandem duplications, suggesting that the former was the main route for expanding *SPL* genes in wheat and that many homologous genes on the different wheat chromosomes support the high conservation of the family.

Ka/Ks, the nonsynonymous to synonymous substitution ratio, determines the selection pressure of duplicated genes. Thus, the protein and CDS sequences of each duplicated gene pair were compared, and Ka/Ks ratios and divergence times were calculated. According to the results (Additional file 1: Table S3), the Ka/Ks ratios of all *TaSPL* gene pairs are< 1, indicating that the evolution of *TaSPL* genes was accompanied by intense purifying selection. We also predicted that the divergence time of *TaSPL* gene pairs to be approximately 27 Mya.

We detected the colinearity of wheat with other species at a genome-wide level to better understand the origin of the *TaSPL* gene family. Most *TaSPL* genes have orthologous genes in rice, maize, *A. tauschii*, and *B. distachyon* (Fig. 4B-4E, Additional file 1: Table S4-S7). We detected 67, 110, 52, and 56 gene pairs, respectively. The Ka/Ks ratios between wheat and rice, maize, *A. tauschii*, and *B. distachyon* were calculated to be 0.38, 0.39, 0.29, and 0.31. All of the collinear gene pairs showed a value lower than 1, confirming that the evolution of the *SPL* gene family in wheat has undergone a strong purifying selection**.** The divergence time of collinear gene pairs in rice was the same as that in maize, at approximately 52 Mya, and earlier than that in *A. tauschii* (39 Mya) and *B. distachyon* (41Mya), indicating that *TaSPL* gene family shares an intimate correlation with those in rice, maize, *A. tauschii*, and *B. distachyon*.

### GO annotation analysis and protein–protein interaction network of *TaSPLs*

**Fig. 5:**
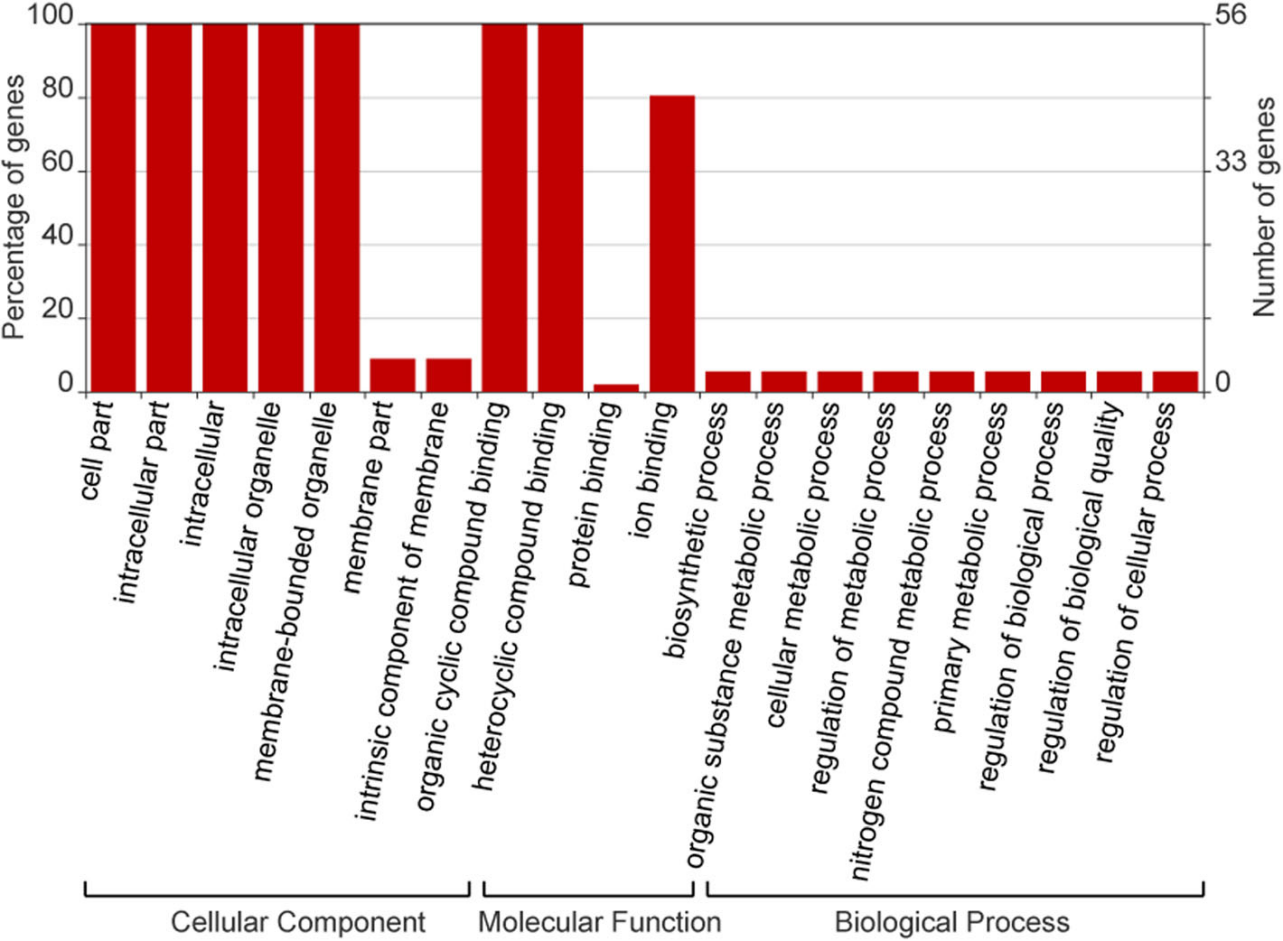
GO annotations for TaSPL proteins. The GO annotation is divided into three main categories: cellular component, molecular function and biological process. The y-axis on the left represents the percentage of genes in each category, and the number of genes is showed on the right

We performed GO annotation analysis on the 56 proteins, revealing that they may participate in a range of cellular components, molecular functions and biological processes (Fig. 5, Additional file 1: Table S8). The 56 TaSPL proteins were assigned a total of 20 GO terms; although only a few GO terms belong to cellular component, many proteins were enriched in this category. Under the cellular component category, the most highly enriched categories are related to cell part, intracellular part, and organelle. All of the TaSPLs can participate in these three processes, whereas less than 30% of TaSPLs are involved in membrane formation. Under molecular function, all proteins have the capacity to bind to other molecules, such as organic cyclic compound binding and ion binding. Among them, 100% of the TaSPLs can bind to heterocyclic compounds and 80% to ions. Regarding biological processes, less than 20% of the TaSPLs participate in biological processes, such as nitrogen metabolism, and regulation of cellular processes.

**Fig. 6:**
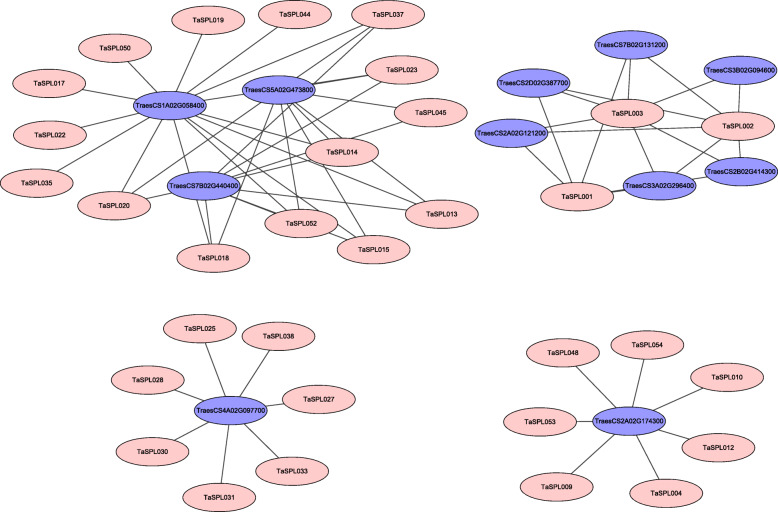
The protein-protein interaction network between TaSPLs and other wheat proteins. The TaSPL proteins are in pink, and the other wheat proteins are in purple

To understand the protein-protein interactions between TaSPLs and other proteins in wheat, we constructed a protein-protein interaction network (Fig. 6, Additional file 1: Table S9). Thirty-two TaSPL proteins and a total of 64 interacting protein branches were detected. Among them, 10% of TaSPLs can interact with at least 6 proteins, such as TaSPL001, TaSPL002, and TaSPL003, suggesting that these 3 TaSPL proteins play a significant role in the regulation of protein networks. The sequences of additional wheat proteins that interact with TaSPL proteins were submitted to the CDD database to predict their conserved domains, which we revealed various transcription factor families, such as AP2, Cu-Zn_Superoxide_Dismutase, and MADS_MEF2_like. Among them, 32 interaction branches were identified as the interaction of TaSPLs with the AP2 family. Therefore, we suggest that highly possible that TaSPL proteins interact with proteins of the AP2 family to regulate wheat development.

### Promoter analysis of *TaSPL* genes

The specific cis-acting elements contained in promoter region can regulate the gene expressions. We analyzed the 1.5-kb DNA sequence upstream of 56 *TaSPL* genes and identify potential cis-acting elements (Figure S1, Additional file 1: Table S10). A variety of cis-elements in the promoter regions of *TaSPL* genes clustered into three subdivisions: hormone and stress response elements, light-responsive elements, and plant growth and development elements. Among the three types, most *TaSPL* genes contain light-responsive elements, such as ACE, Sp1, and G_box. In particular, the G_box was found in 60% (34) of the *TaSPL* genes, suggesting that *TaSPL* expressions may be regulated by light and that the G_box is the most important for wheat development. In addition, the ABRE element, which is associated with abscisic acid responsiveness were distributed widely throughout 80% of the *TaSPL* genes, suggesting that *TaSPLs* are associated with regulation of the ABA metabolic pathway. At the same time, some elements related to plant growth and development, such as CAT_box, O2_site, and HD_ZIP 1, were found in *TaSPL* genes, indicating that the *TaSPL* gene family plays roles in regulating physiological processes.

### Expression patterns of *TaSPL* genes in different wheat tissues

**Fig. 7:**
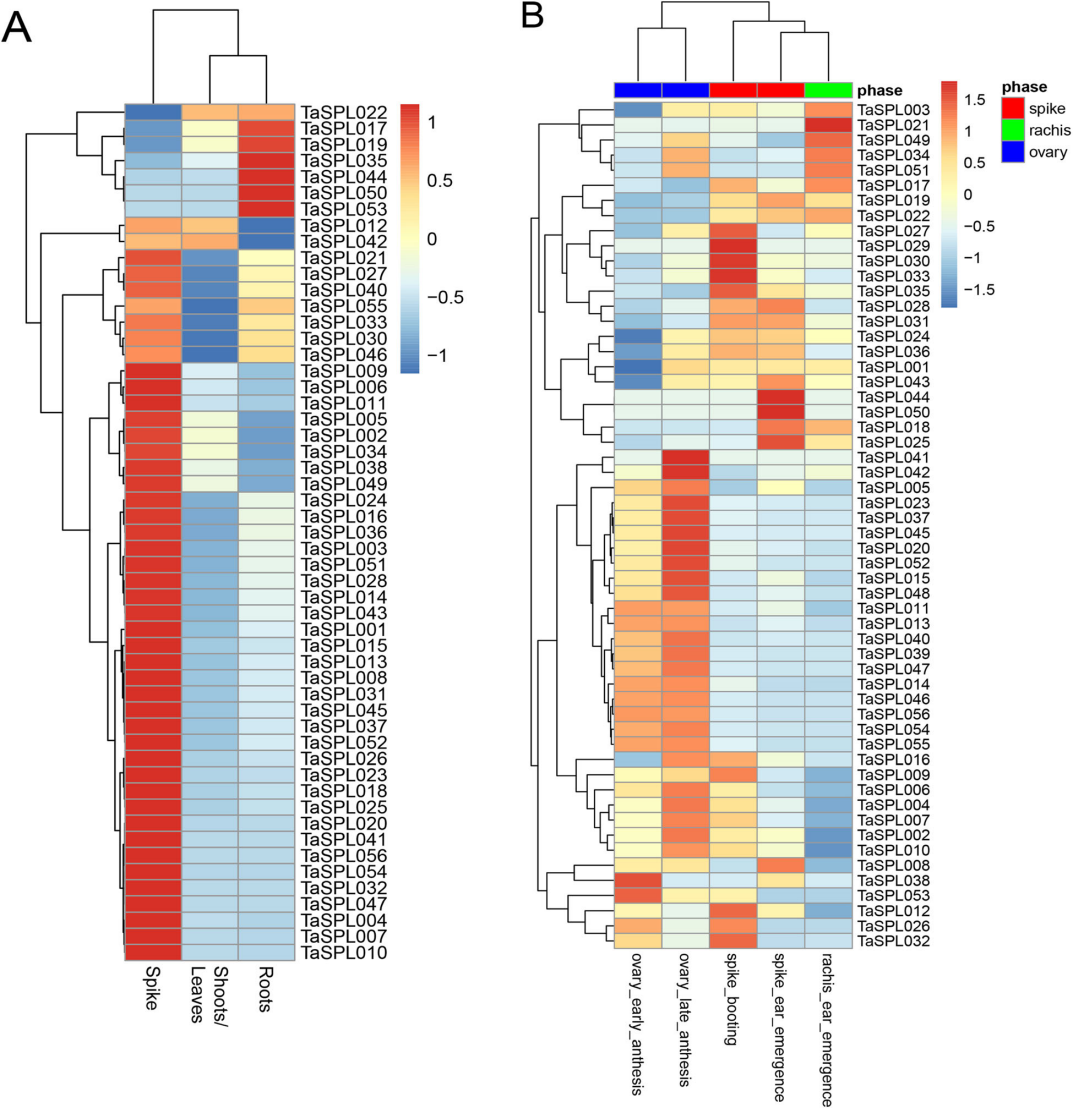
Expression patterns of *TaSPL* genes. Expression profiling of 53 *TaSPL* genes in roots, shoots/leaves and spike (**a**). Expression profiling of 56 *TaSPL* genes in 5 organs, including ovary_early_anthesis, ovary_late_anthesis, spike_booting, spike_ear_emergence, and rachis_ear_emergence (**b**). High expression levels are shown in red, and low expression levels are shown in blue

The transcriptional data were downloaded from the wheat expression browser website to determine the tissue-specific expression patterns of *TaSPL* genes (Fig. 7A). The results showed that 53 *TaSPLs* exhibited different expression levels in shoots/leaves, roots, and spikes of wheat. For example, 46 *TaSPLs* displayed high expression in spikes, and 7 genes were found to be highly expressed in roots, implying that *TaSPLs* are primarily expressed in spikes and participate in the development of spikes. In addition, genes in the same subgroup shared similar expression patterns. All *TaSPL* genes in subfamily V showed high expressions in roots and expressions of subfamily VIII members in shoots/leaves, and roots were observed to be low, revealing that *TaSPL* in the same family share conserved functions.

Expressions of *TaSPL* genes in five organs (ovary_early_anthesis, ovary_late_anthesis, spike_booting, spike_ear_emergence, and rachis_ear_emergence) were also analyzed (Fig. 7B). The 56 genes were expressed as follows: 75% (42) were highly expressed in ovary_anthesis, 55% (31) were highly expressed in spikes, and only 14% (8) showed high expressions in rachis ear emergence. These findings suggest that *TaSPL* genes are primarily expressed in organs related to flower development. Among them, 55% (32) were mainly found in ovary_early_anthesis. Additionally, 68% (38) showed high expression in ovary_late_anthesis, especially, 12 *TaSPL* genes, such as *TaSPL005*, *− 038*, and *− 052*, expressed at higher levels in ovary_anthesis, suggesting that *TaSPL* genes are involved in the development of wheat flowers and that 12 *TaSPL* genes are essential for flower development. Moreover, 46% (26) of the *TaSPLs* displayed elevated expressions in spike_booting, and 29% (16) were expressed at higher levels in spike_ear_emergence. Therefore, we inferred that *TaSPL* genes are related to the development of flowers and spikes, influencing the formation of flowers and spikes by participating in the development of anthers and spikes.

### Quantitative RT-PCR analysis of 21 *TaSPL* genes

**Fig. 8:**
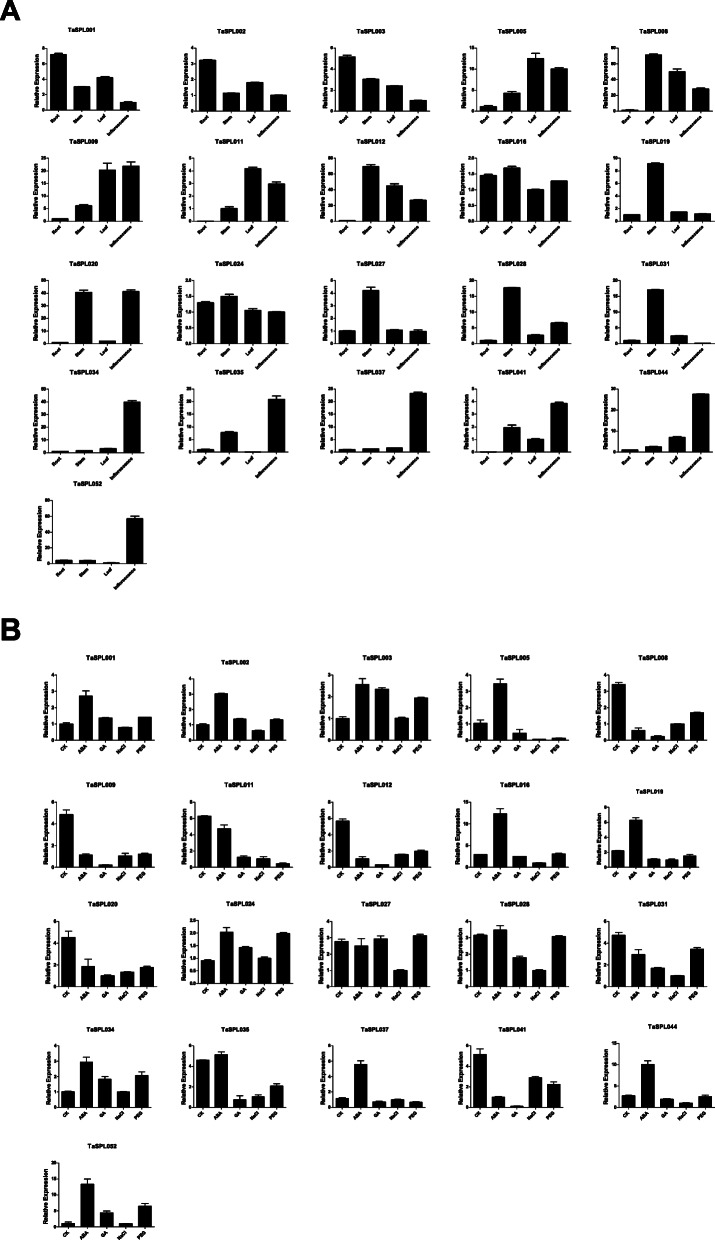
Quantitative RT-PCR analysis of 21 *TaSPL* genes. Relative expression levels of 21 genes in 4 tissues (roots, stems, leaves and inflorescence) (**a**); relative expression levels of 21 genes under 4 different treatments (ABA, GA, NaCl and PEG) (**b**)

To further investigate the possible functions of *TaSPLs*, qRT-PCR was used to measure the expression patterns of 21 randomly selected *TaSPL* genes in four tissues (roots, leaves, stems, and inflorescences) (Fig. 8A). Twenty-one *TaSPLs* were expressed in the four tissues, with strong tissue-specific expression patterns. The number of *TaSPL* genes that were highly expressed in inflorescences and stems was higher than that in roots and leaves. For example, 8 genes (*TaSPL009*, *− 020*, *− 034*, *− 035*, *− 037*, *− 041*, *− 044*, −and *− 052*) showed high expression levels in inflorescences, and 8 genes (*TaSPL008*, *− 012*, *− 016*, *− 019*, *− 024*, *− 027*, *− 028*, and *− 031*) were expressed in stems; however, only 3 (*TaSPL001*, *− 002*, and *− 003*) were mainly found in roots, and 2 (*TaSPL005*, and *− 011*) showed high expressions in leaves. This suggests that *TaSPL* genes are primarily expressed in inflorescences and stems and play critical roles in the development of wheat flowers and stems.

As *SPL* genes may be associated with plant stress, hormone responses and other signaling processes, the expression levels of 21 *TaSPLs* under 4 treatments were analyzed (Fig. 8B). Compared to CK, 13 genes, such as *TaSPL001*, *− 002* and − *019* were upregulated by ABA treatment, suggesting that *TaSPL* genes are involved in the ABA pathway. The expression levels of more than half of the genes decreased gradually under the other 3 treatments. For example, 14 genes, such as *TaSPL009*, *− 005*, and *− 008* was downregulated by GA treatment; 19 genes, such as *TaSPL011*, *− 012*, *− 016*, and *− 019* was downregulated by salt treatment; and 14 genes were downregulated by PEG treatment. These results indicate that *TaSPL* genes play negative roles under salt, GA and PEG treatments.

## Discussion

### Characteristics of *SPL* family genes in wheat

The *SPL* genes encode plant-specific zinc finger proteins play critical roles in regulating plant growth and development. These genes have been identified in many green plants, such as rice [27], *A. thaliana* [10], maize [28], and buckwheat [29]. Wheat is a major crop, and the complete genome sequencing of Chinese Spring would provide detailed information to study *SPL* gene families. In this study, we used comparative genomics methods to examine 56 *TaSPLs*, 13 *TuSPLs*, 17 *AetSPLs*, and 17 *BdSPLs*. The number of *SPL* genes in *T.urartu*, *A. tauschii* and *B. distachyon* is similar to that in *A. thaliana* (16) [10], and rice (19) [27] but lower than that in maize (31) [28] and buckwheat (24) [29]. However, the number of *TaSPLs* is higher than that of the above species; in particular, there are threefold as many *TaSPLs* than *TuSPLs* and *AetSPLs*. This relationship of *TaSPL* with *TuSPL* and *AetSPL* is consistent with previous studies on the *Prx* and *PK* gene family in wheat, *T.urartu*, and *A. tauschii* [30]. It is that wheat is allohexaploid, whose origin involved two polyploidization events, and it appears that many gene duplication events occurred in the wheat genome, allowing the number of *TaSPLs* to increase compared to other species. All TaSPL proteins were found to localize to the nucleus; however, during the evolution process of the *TaSPL* gene family, the insertion or loss of exon, and functional diversity and sub-functionalization of SPL proteins in different subfamilies caused a large difference in amino acid length and type. Therefore, the isoelectric points and MWs of TaSPL proteins are significantly different. This result agrees with that for other grass crops, indicating that the *SPL* genes are conserved among species.

### Evolution of *SPL* family genes in wheat

It is suggested that *SPL* genes existed only in green plants and predated the divergence of the green algae [8]. The ancestor *SPL* originally formed into two different lineages in land plants, named clade I and clade II. The clade I has conserved structure characteristics that possess more exons and longer protein sequences [31]. Our results showed that all *SPL* genes from wheat contained 1–11 exons. The number of exons in most *SPL* genes was 3, with only two subgroups (VIII and X) having more than 10 exons. Furthermore, the protein sequences of TaSPLs in subgroup VIII and X are greater than TaSPL proteins in the remaining subgroups. We inferred that *TaSPL* genes in subgroup X and VIII belong to clade I and that the remaining *TaSPL* genes belong to clade II. In accordance with Zhang’s [32] study, we found that the means of Ka and Ks for subgroup X and VIII were lower than those for the other subgroups. Further analysis revealed that subgroup VIII and X genes have lower Ka/Ks ratio (0.25) compared to other duplicated genes. We consider that the evolutionary mechanism of the *TaSPL* gene family is similar to that in other land plants can be clustered into two clades and the subgroup VIII and X genes have evolved slower than other genes.

To explore the evolutionary and functional divergence of the *SPL* gene family in wheat, we identified and determined the phylogenetic relationships of this family. Seventeen orthologous gene pairs of *TaSPL* were detected in this phylogenetic tree, including 13 gene pairs from wheat orthologous with *A. tauschii*, 3 gene pairs orthologous with *T.urartu*, and 1 gene pair orthologous with barley. No gene pair orthologous with rice, *A. thaliana* or *B. distachyon*, suggesting that *TaSPLs* have a close relationship with *SPL* genes in *A. tauschii* and *T.urartu*. From gene duplication results, 81 *TaSPL* gene pairs are duplicated genes and the high levels of collinear gene pairs were observed among rice, maize, *A. tauschii*, and *B. distachyon*. The number of segmental duplication events was greater than that of tandem duplication events, indicating that segmental duplication contributed to *TaSPL* genes expansion. Gene duplication and syntenic analysis confirmed that no positive selection occurred in *TaSPLs*, additionally, *TaSPL* genes underwent strong purifying selection. Our results are consistent with the study of *SPL* genes in buckwheat [29] and rice [31], indicating that the evolution of *TaSPL* genes is comparable with that of other plants.

In brief, we inferred that the evolution of the *SPL* gene family in wheat is similar to that in other land plants. Compared with *A. thaliana* and *B. distachyon*, *TaSPL* genes share a strong relationship with *AetSPLs* and *TuSPLs*.

### Expression patterns and potential function of the *SPL* gene family in wheat

In general, genes perform a function based on their expression in the organism, and the expression patterns of genes reflect the gene functions [13]. In this study, we examined the expression patterns of 21 *TaSPL* genes, with 38% showing high expression in stems and inflorescences, implying *TaSPL* genes mainly perform functions in wheat inflorescences and stems. In *A. thaliana*, Chao [33] reported that the *AtSPL1* and the *AtSPL012* showed higher expression levels in inflorescences, overexpressed the two genes enhanced the inflorescence thermotolerance. Xu [10] found that *AtSPL2*, *− 9*, *− 10*, *− 11*, *− 13*, and *− 15* play dominant roles in the transformation of plants from vegetative to reproductive stages. Except *AtSPL10*, the remaining 5 genes promote both floral meristem identity and floral induction. Homologous genes may share similar functions. The results of evolutionary analysis showed that *AtSPL1* and *AtSPL12* are highly orthologous to *TaSPLs* in the VIII subgroup, including, *TaSPL034*; *AtSPL9*, and *− 15* share close relationships with *TaSPL044* and *− 035*. *AtSPL13* is orthologous to *TaSPL* genes belonging to subfamily IV, such as *TaSPL020*, *− 037* and *TaSPL052*. These 6 *TaSPL* genes were expressed at higher levels in inflorescences. We inferred that these genes are involved in the development of inflorescences. Three genes (*TaSPL001*, *− 002*, *− 003*) containing 10 exons were expressed at higher levels in wheat roots than in three other tissues. However, the remaining genes, containing fewer or more than 10 exons, have lower expression in roots. Therefore, we inferred that these 3 *TaSPL* genes are essential for the development of roots and the insertion of exons will affect *SPL* gene expressions in wheat tissues.

Function analyses have uncovered that the *SPL* gene family governs many aspects of plant growth and development in environmental stresses [32]. In this study, we found that expression of *SPL* genes was low in the seedling stage under 4 treatments. Wu [34, 35] and Wang [36] reported that miR156 presents the highest expression levels at seedling stage. As the plant progresses from a seedling to more mature stages, its expression gradually decreases. In contrast, the expression levels of the target genes *AtSPL3* and *AtSPL9* were lower in the seedling stage and gradually increased throughout the vegetative growth stage. Therefore, we deduced that miR156 inhibits the expression of *TaSPL* genes of the seedlings stage. In our study, we found 21 *TaSPL* genes were up- or downregulated by 4 different treatment, with 13 *TaSPL* genes being upregulated by ABA, most of them contained the ABRE element in their promoters, suggesting a positive role in ABA-mediated plant growth. Fourteen *TaSPL* genes were downregulated by GA, suggesting that these 14 genes play a negative role in GA-mediated plant growth. Cui [37] reported that *AtSPL9* was downregulated by salt and drought treatments, regulating *A. thaliana* flowering, implying that *AtSPL9* responds to these two stress treatment. We found that *TaSPL035* and *− 044*, which were downregulated by NaCl and PEG treatments, share high homology with *AtSPL9*, suggesting that these two genes can respond to salt and drought stress. In brief, we inferred that the *TaSPL* gene family plays critical roles in wheat development, especially, this family is involved in stems and flowers development, and that members of the family can participate in abiotic stress.

## Conclusions

Phylogeny and diversification of *SPL* genes in wheat were investigated from different levels, including gene structures, evolutionary relationships, synteny analyze, GO annotation, protein-protein interaction, and expression patterns. The *SPL* gene family in wheat was expanded by segmental duplication and purifying selection, during the evolution. All 56 *TaSPL* genes were divided into 8 subgroups, genes in same subgroup share similar evolutionary features and expression patterns, implying similarity function potentially of *TaSPL* genes. Twenty-one gene expressions were detected and that the *TaSPL* genes have tissue-specific expression patterns in different tissues and these genes exhibited higher expression levels at stems and other tissues related to spike development, such as anthers and inflorescence, suggesting that *TaSPL* genes regulate the development of stems and influence the formation of flowers and spikelets. Here, we first reported the identification, phylogenetic, and expression analysis of the *TaSPL* gene family, which will serve as a foundation for further elucidation of the potential biological functions of *SPLs* in plants.

## Methods

### Identification of *SPL* genes in wheat

Whole-genome data of wheat were obtained from the Ensembl plant database (http://plants.ensembl.org/info/website/ftp/index.html), and the SBP domain (PF03110) was downloaded from the PFAM database (https://pfam.xfam.org/). Then, the SBP domain was used as the query sequence to find proteins containing an SBP domain in wheat using the HMMER 3.0 program with the threshold of e < 1e^− 5^ [38]. The SBP protein sequences from rice [27] and *A. thaliana* [10] were used as query sequences to search against the wheat protein dataset using the BLASTP program, and the threshold was set at e < 1e^− 5^ and 50% identity. Putative wheat *SPL* genes were preliminarily identified by analyzing the results from HMM and BLASTP. Then, the NCBI-CDD web server (https://www.ncbi.nlm.nih.gov/Structure/bwrpsb/bwrpsb.cgi) and the PFAM database were used to further confirm the candidate *SPL* genes of wheat. The protein sequences of *TaSPLs* were computed in the ExPASy server (https://web.expasy.org/compute_pi/) [39] to obtain the theoretical isoelectric point (PI) and molecular weight (MW), and the cello web server (http://cello.life.nctu.edu.tw/) [40] was used to predict the subcellular localization of these proteins.

### Chromosomal location, gene structure and conserved motif analysis of *TaSPL* genes

Physical locations of *TaSPL* genes were obtained from the genome annotation information (gff3) of wheat, and the *TaSPL* genes were mapped to the wheat chromosomes. The structure of genes and coding sequences (CDS) of *TaSPLs* were analyzed to investigate the exon-intron organization, and the results were displayed in Gene Structure Display Serve (http://gsds.cbi.pku.edu.cn/) [41]. Conserved motifs of *TaSPL* proteins were identified using MEME v4.9.0 (http://meme-suite.org/tools/meme) with the optimum motif set at ≥10 and ≤ 200 amino acids and the maximum number of motif set at 20 [42].

### Phylogenetic analyses

SPL protein sequences of wheat, *B. distachyon*, *A. tauschii*, *T.urartu*, rice, and *A. thaliana* were used to perform the phylogenetic analysis. ClustalX 2.0 software [43] with the default parameters was utilized to conduct multiple sequence alignment. An un-rooted neighbor joining (NJ) tree was constructed using MEGA 6.0 [44] software with 1000 bootstrap replications.

### Gene duplication and synteny analysis of wheat with rice, maize, *A. tauschii*, and *B. distachyon*

MCScanX [45] software was used to detect collinear regions between *TaSPL* genes, as well as collinear blocks of *TaSPL* genes with 4 other species (*B. distachyon****,*** rice, *A. tauschii*, and maize). Gene duplication events of *TaSPLs* and synteny relationships between the aforementioned species were visualized using the Circos 0.67 tool [46]. CDSs and protein sequences of collinear gene pairs were compared, and KaKs ratios were calculated using KaKs_Calculator software [47]. Finally, the divergence time of collinear gene pairs was calculated using the method of Wang [T = Ks/(2λ × 10^− 6^) Mya (λ =6.5× 10^− 9^)] [3].

### GO annotation and protein-protein interaction network analysis of TaSPLs

GO annotation of SPL proteins in wheat was available from the PLAZE database (https://bioinformatics.psb.ugent.be/plaza/versions/plaza_v4_monocots/) [48] and the Plant Transcriptional Regulatory Map database (http://plantregmap.gao-lab.org/). The GO annotation results were visualized using the WEGO (http://wego.genomics.org.cn/) online tool [49].

Based on orthogonal genes between wheat and *A. thaliana*, the ArenaNet V2 tool [50] and the String database (https://string-db.org/cgi/input.pl) [1] were used to construct an interaction network between TaSPL proteins and other wheat proteins. A trusted value of > = 0.8 in the String database was used to confirm the interaction network, which was graphically displayed by Cytoscape software [51].

### Promoter analysis of TaSPLs

The upstream 1.5-kb DNA sequences of the *TaSPL* genes were downloaded from the Ensembl plant database, and then submitted to the PLACE database (http://bioinformatics.psb.ugent.be/webtools/plantcare/html/) to predict cis-regulatory elements in promoter regions [40].

### Gene expression, materials and qRT-PCR analysis of *TaSPLs*

The transcriptional data of *TaSPL* genes in 3 tissues (roots, shoots/leaves, spike) and 5 organs (ovary_early_anthesis, ovary_late_anthesis, spike_booting, spike_ear_emergence, and rachis_ear_emergence) of wheat were obtained from wheat expression browser website (http://www.wheat-expression.com/download) to detect the expression profile [52]. R was used to display the expression patterns in a heat map.

For qRT-PCR, the Chinese Spring cultivar of wheat was grown in a greenhouse in 2019. Roots, leaves, stems, and inflorescences were collected during the heading stage. Three-week-old seedlings were exposed to 200 mM NaCl, 100 μM gibberellin acids (GA), 100 μM abscisic acid (ABA), or 20% PEG-600 for 2 h, and samples were then collected. All samples were stored at − 80 °C for RNA extraction using RNAiso Reagent (TaKaRa, Beijing, China) with three biological replicates. cDNA was synthesized using the RT Master Mix Perfect Real-Time kit (TaKaRa, Beijing, China). QuantStudio™ Real-Time PCR software was used to perform qRT-PCR, and the 2^(−ΔΔCt)^ analysis method was used to determine the relative expression levels of 21 randomly selected *TaSPLs*. We chosed *TraesCS6B02G243700* as the reference gene to normalize the expression levels of the *TaSPL* genes. The primers used are listed in Additional files 1: Table S2.

## Supplementary information

**Additional file 1: Table S1.** Characteristic features of the *SPL* gene family identified in wheat. **Table S2.** Primers used for qRT-PCR. **Table S3.** Ka/Ks ratios and estimated divergence time for duplicated *TaSPL* gene pairs. **Table S4.** Ka/Ks ratios and estimated divergence time for orthologous *TaSPL* genes between wheat and rice. **Table S5.** Ka/Ks ratios and estimated divergence time for orthologous *TaSPL* genes between wheat and maize. **Table S6.** Ka/Ks ratios and estimated divergence time for orthologous *TaSPL* genes between wheat and *A. tauschii*. **Table S7.** Ka/Ks ratios and estimated divergence time for orthologous *TaSPL* genes between wheat and *B. distachyon*. **Table S8.** GO annotations of TaSPL proteins. **Table S9.** The protein-protein interaction between TaSPLs and other proteins in wheat. **Table S10.** Cis-elements contained in the *TaSPL* genes promoter region. **Figure S1.** Cis-regulatory elements in the promoter region of *TaSPL* genes.

## Data Availability

The Chinese Spring cultivar used in the experiment is supplied by Professor Ma Lingjian of Northwest Agricultural and Forestry University. The datasets supporting the results of this article are included in the article and Additional files.

## Abbreviations

TaSPL: Wheat SPL
ABA: Abscisic acid
GA: Gibberellic acid
CDS: Coding sequence
HMM: Hidden Markov Model
qRT-PCR: Quantitative real-time polymerase chain reaction
