# Supplementary figures and images for: Genome-wide identification, phylogeny and expression analysis of the SPL gene family in wheat
Source: BMC Plant Biol. 2020 Sep 11;20:420. doi: 10.1186/s12870-020-02576-0 (PMC7488452; doi:10.1186/s12870-020-02576-0)

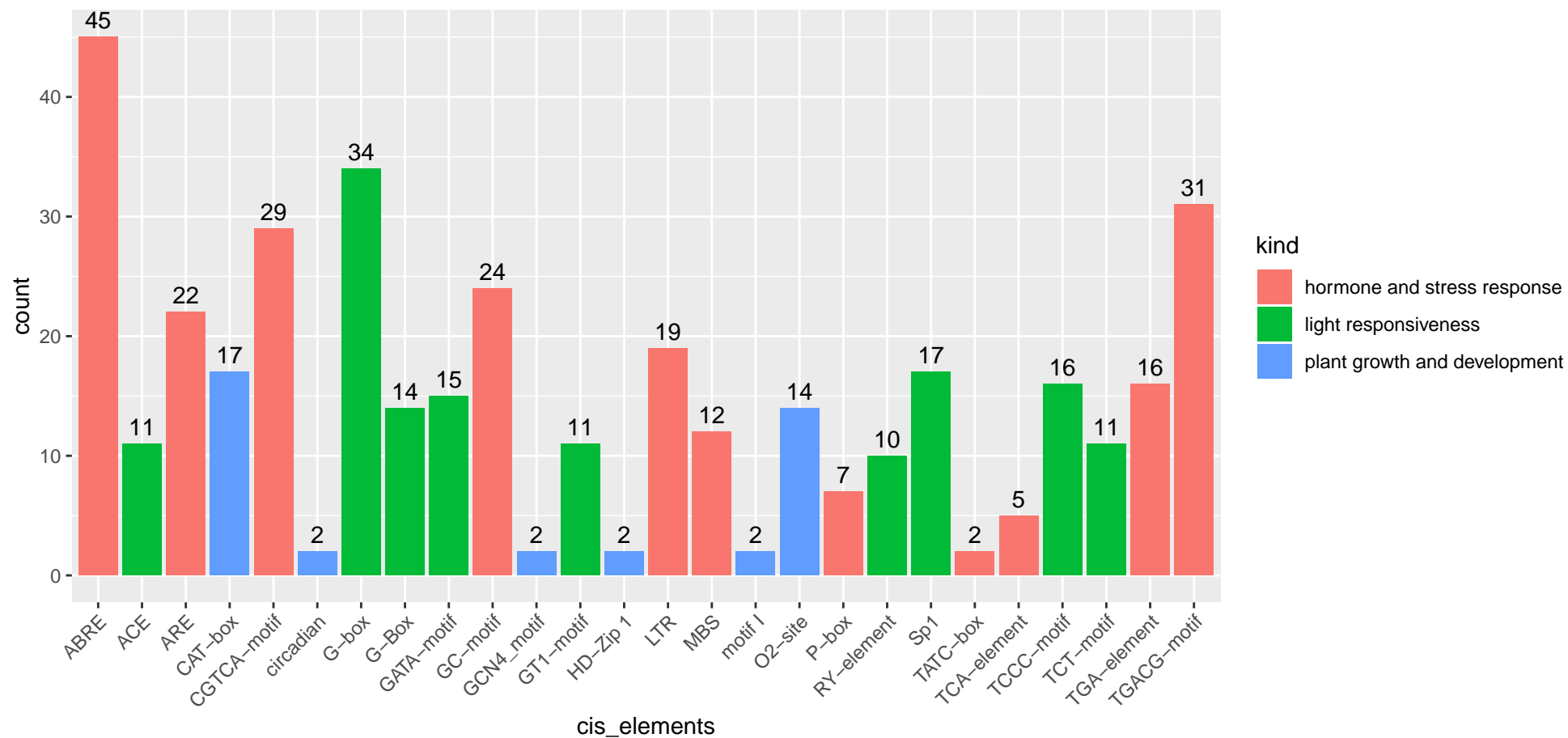

Supplement: Supplementary file 1 — Additional file 1: Table S1. Characteristic features of the SPL gene family identified in wheat. Table S2. Primers used for qRT-PCR. Table S3. Ka/Ks ratios and estimated divergence time for duplicated TaSPL gene pairs. Table S4. Ka/Ks ratios and estimated divergence time for orthologous TaSPL genes between wheat and rice. Table S5. Ka/Ks ratios and estimated divergence time for orthologous TaSPL genes between wheat and maize. Table S6. Ka/Ks ratios and estimated divergence time for orthologous TaSPL genes between wheat and A. tauschii. Table S7. Ka/Ks ratios and estimated divergence time for orthologous TaSPL genes between wheat and B. distachyon. Table S8. GO annotations of TaSPL proteins. Table S9. The protein-protein interaction between TaSPLs and other proteins in wheat. Table S10. Cis-elements contained in the TaSPL genes promoter region. Figure S1. Cis-regulatory elements in the promoter region of TaSPL genes. [file 12870_2020_2576_MOESM1_ESM.zip › Figure S1.pdf]
